# Swedish Genome-Wide Haplotype Association Analysis Suggests Breast Cancer Loci with Varying Risk-Modifying Effects

**DOI:** 10.3390/genes15121616

**Published:** 2024-12-17

**Authors:** Litika Vermani, Elin Barnekow, Wen Liu, Camilla Wendt, Per Hall, Sara Margolin, Annika Lindblom

**Affiliations:** 1 Department of Molecular Medicine and Surgery, Karolinska Institutet, 17176 Stockholm, Sweden; litika.vermani@ki.se (L.V.); wen.liu@neuro.uu.se (W.L.); 2 Department of Clinical Science and Education, Södersjukhuset, Karolinska Institutet, 11883 Stockholm, Sweden; elin.barnekow@regionstockholm.se (E.B.); camilla.wendt@regionstockholm.se (C.W.); per.hall@regionstockholm.se (P.H.); sara.margolin@regionstockholm.se (S.M.); 3 Department of Oncology, Södersjukhuset, 11883 Stockholm, Sweden; 4 Department of Neuroscience, Uppsala University, 75237 Uppsala, Sweden; 5 Department of Medical Epidemiology and Biostatistics, Karolinska Institutet, 17165 Stockholm, Sweden; 6 Department of Clinical Genetics, Karolinska University Hospital, 17164 Stockholm, Sweden

**Keywords:** breast cancer, GWAS, haplotype analysis, modifying effect, genetic risk loci

## Abstract

**Background:** To find support for risk-modifying genes in breast cancer, a haplotype GWAS in sporadic breast cancer cases was undertaken. The results were compared with the results from previous analyses in familial cases and all cases from the same Swedish cohort. **Methods:** In total, 2550 women with sporadic invasive breast cancer and 5021 healthy controls were included in a sliding-window haplotype GWAS using PLINK 1.07. **Results:** The analysis of sporadic cases confirmed the loci on chromosomes 10q26.13, 11q13.3, and 16q12.1 and suggested one novel locus on chromosome 12p11.21 (OR = 1.42 *p* = 4.55 × 10^−8^). A comparison between these loci and the same loci in the analyses of familial cases and all breast cancer cases was undertaken. **Conclusions:** Haplotype GWAS in sporadic cases of Swedish breast cancer cases supported known risk loci and suggested another risk locus. The loci identified in the analysis of sporadic and all breast cancer cases were suggested to act as modifiers of the risk of breast cancer. Haplotype analysis identified other loci with higher odds ratios than single-variant analysis. Further studies are needed to find out how to best include the findings in breast cancer prevention.

## 1. Background

In Western countries, breast cancer (BC) is one of the most diagnosed cancers among women. BC is the most prevalent cancer among women in Sweden, with 7452 new cases in 2022, accounting for 23.7% of cancers among females (GLOBOCAN 2022) [1]. High risk of the development of BC due to rare pathogenic variants in known BC genes accounts for less than 5% of BC cases in the general population [2,3]. Moderately penetrant genes that are inherited and contribute to BC susceptibility explain approximately 30% of heritability in familial BC cases [4,5,6]. Over the past decade, different GWAS have suggested several cancer susceptibility loci [7,8,9]. Most of the heritability remains unexplained. 

We have previously published two haplotype GWAS in two Swedish BC cohorts from BCAC, KARMA and KARBAC [10,11]. Both studies used haplotype analysis instead of SNP analysis. Haplotype analysis has been used several times and been able to result in more exact location for risk loci, as well as higher ORs, compared to published GWAS. Published GWASs typically used SNPs, single variants, to analyze cases and controls, while haplotype analysis analyzed estimated haplotypes based on the available genomic data in the study population (cases and controls). The first analysis of all BC cases gave strong support for the three strongest loci on chromosomes 10q26.13, 11q13.3, and 16q12.1, and revealed sub-loci within these three loci [10]. In addition, a novel locus on chromosome 8p21.1 was found [10]. In the analysis of the familial cases, the same three common loci were supported, plus five new ones on 9p24.3, 11q22.3, 15q11.2, 16q24.1, and Xq21.31 [11]. The odds ratios (ORs) were much higher in the familial analysis, supporting the hypothesis of an overrepresentation of cases with stronger genetic contribution. 

In a previous GWAS study of colorectal cancer, familial, sporadic, and all CRC cases were studied separately and published together at the same time [12]. This study had 2179 sporadic, 484 familial, and thus total 2663 CRC cases. No statistically significant results were obtained in the analysis of all 2663 CRC cases and only one locus was found in the 484 familial cases where the high OR, above four, resulted in statistical significance [12]. However, the result from the analysis of the 2179 sporadic cases unexpectedly resulted in two statistically significant loci [12]. Both those ORs were slightly higher compared to the ORs for the same haplotypes in the analysis of all 2663 cases [12]. Thus, these two loci were interpreted as modifier loci, and that the effect on the risk was strongly imposed by the risk from environmental exposure and less from genetics.

Therefore, we aimed to perform a haplotype GWAS in the sporadic cases from the same two BCAC cohorts studied before (KARMA and KARBAC), and to compare the results with the analyses of familial and all cases. The hypothesis was that we could find support for modifying genes in BC.

## 2. Material and Methods

### 2.1. Cases and Controls

The cases and controls used in this study were from two Swedish cohorts, KARMA and KARBAC. The total number of cases of invasive BC was 3215—KARMA (*n* = 2712) and KARBAC (*n* = 503)—and the controls were collected only from KARMA and included 5032 healthy individuals without any family history of BC [13,14,15]. Family history was defined as at least one first-degree relative with BC. Thus, sporadic cases were defined as cases without a first-degree relative with BC. The cohorts used in the current study have been described previously [13,14,15]. All the participating individuals gave written informed consent, and the studies were approved by the local ethical board (KARMA: approved by the Ethical Committee of the Karolinska Institute, Dnr 2010/958-31/1; KARBAC1: approved by the Ethical Committee of the Karolinska Institute, Dnr 98-232; KARBAC2: Dnr 2011/1686-32 and 2012/1453-32). For the subgroup analysis on sporadic BC, we used 2563 BC cases; for familial BC, we used 652 cases and 5032 healthy controls. 

### 2.2. Genotyping and Quality Control

Illumina Infinium OncoArray-500K B BeadChip was used for genotyping 7595 individuals (3215 BC cases and 5032 healthy controls) [7,16]. The two cohorts shared a total of 474,706 SNPs. PLINK v.1.9 was used to merge the datasets [17,18]. TOP strand format was taken into consideration, while quality control (QC) variants were excluded if call rates were <98% (2332 variants removed), if there was a <0.01 minor allele frequency (138,834 markers removed), or if there was deviation from Hardy–Weinberg equilibrium at *p* < 0.001 (634 markers removed). All the details regarding the QC and MDS analysis are already described in the previous study [10]. After quality control, 332,906 SNPs, 2550 sporadic cases, and 5021 controls remained for statistical analysis.

### 2.3. Statistics

A sliding-window haplotype GWAS using PLINK 1.07 was performed [19,20]. This haplotype analysis was performed with high-performance computers for big data analysis in Uppsala Multidisciplinary Center for Advanced Computational Science (UPPMAX). As part of the sliding-window method, a window sliding over a size of 1 to 25 SNPs was used for the haplotype GWAS. All possible haplotypes from the 1st SNP to the 25th SNP were tested in this analysis, and this generated several haplotypes of differing lengths for the same locus. A logistic regression model was used to examine the effect of an SNP or a haplotype of varying length on the risk of sporadic BC [19,20]. The default setting of minor haplotype frequency of 0.01 in PLINK v.1.07 was applied. This means that each haplotype or SNP with a frequency above 1% was tested individually against all other SNPs/haplotypes with frequencies above this threshold [19,20]. The following parameters were included as part of the haplotype analysis: hap-logistic (haplotype imputation based on multi-marker predictors), sliding hap-window of 1–25 (fixed number of SNPs in the sliding-window model), minor allele frequency (MAF) of 0.01 (variants below the MAF threshold are filtered), and maximum per-SNP missing, geno 0.1 (all variants with missing call rates exceeding the default value 0.1 are removed).

## 3. Results

A haplotype GWAS with 2550 sporadic BC cases and 5021 healthy controls was undertaken. A sliding-window approach examined fixed-size windows of SNPs across the genome and examined windows across 1 to 25 SNPs at a time and accessed whether that window was associated with genetic risk. In the analysis, several overlapping haplotypes of varying lengths and significance were generated. The entire genome was examined with this overlapping window analysis. The analysis of sporadic BC cases resulted in statistical significance (*p* < 5 × 10^−8^) for the same three loci as seen first in BCAC on chromosomes 10q26.13, 11q13.3, and 16q12.1 (two haplotypes), and in the haplotype analysis of all and familial Swedish BC cases in the past [10,11]. The analysis of sporadic cases also suggested one novel locus on chromosome 12p11.21 (OR = 1.42 *p* = 4.55 × 10^−8^) (Supplemental Table S1) (Table 1).

A comparison was made for statistically significant loci in the analysis of sporadic BC (Table 2). Of the five statistically significant results from this study, four were known [10,11] and statistically significant in all and familial BC cases from the same cohorts, KARMA and KARBAC (Table 2). The new locus on chromosome 12p11.21 in the analysis of sporadic BC had a higher OR and therefore had a lower *p*-value compared to the results of the analysis in all BC cases. The results from the same locus in familial BC was not statistically significant (>0.05) (Table 2). The OR for the other loci in the analysis of sporadic BC was lower than in the analysis of familial and all BC cases. 

**Figure 1:**
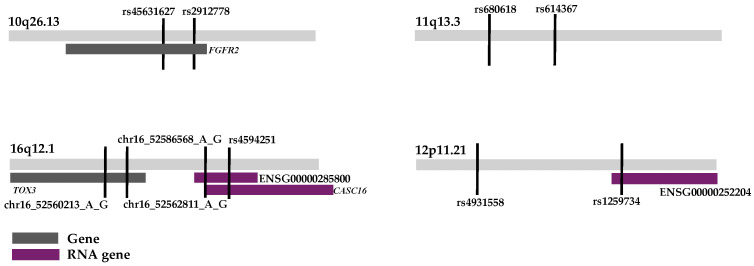
Figure showing each statistically significant locus and associated genes.

Two of the haplotypes were associated with genes. The haplotype region 10q26.13 was within the gene *FGFR2* (Figure 1). The haplotype region 16q12.1 was within the gene *TOX3* (Figure 1). The haplotype on locus 12p11.21 had a small part of an RNA gene in the end of the haplotype region (Figure 1). The second haplotype region on chromosome 16q12.1 overlapped two RNA genes (Figure 1). The haplotype region on chromosome 11q13.3 was not associated with any gene or element (Figure 1).

## 4. Discussion

There are many known risk factors for BC, both genetic and environmental. Genetic risk factors have often been considered the most important considering the known high-risk genes such as *BRCA1*, *BRCA2*, *TP53*, *PALB2*, *CDH1*, *PTEN*, and *STK11* and those with a more moderate risk (*ATM*, *BARD1*, *CHEK2*, *RAD51C*, and *RAD51D*) [21,22]. However, certain genes have also been considered to act as modifiers of risk caused by environmental factors [23].

Here, two cohorts of Swedish BC patients and controls from BCAC were used in a GWAS to specifically search for loci genetically modifying the risk of BC. A family history of BC was used to categorize sporadic, familial, and all BC cases. The hypothesis was that the analysis of sporadic cases should result in higher ORs at loci where the risk is explained by a modifying effect of BC risk caused by certain other risk factors. Similarly, the analysis of familial cases should result in higher ORs compared to the same loci in the analysis of sporadic and all BC patients for loci with a high genetic risk. 

Relating to the hypothesis of this study, the results suggested a modifying effect at loci where the analyses in sporadic BC cases had higher ORs compared to the same loci in all BC cases. A less modifying (and stronger genetic) effect was suggested at risk loci where ORs in all BC cases were higher than those in sporadic cases. In this GWAS in sporadic BC cases, only one novel locus on chromosome 12p11.21 was found (Table 1). This locus was not statistically significant in the previous analyses of familial or all BC cases (Table 2) [10,11]. The novel locus on chromosome 12p11.21 had the highest OR in the analyses of sporadic cases, whilst this value was lower in all cases and the lowest in familial BC, suggesting it to act as a modifying risk locus (Table 1 and Table 2). 

The increased risks estimated from ORs in the analysis of sporadic and all BC samples were similar (Table 2). Thus, statistically significant loci in both sporadic and all BC cases were mostly considered as modifying risk loci, with the highest risk in those exposed to certain environmental risk factors. Risk factors such as diet, lifestyle factors, endocrine disruptors, and exposure to radiation could interact with genetic predispositions in high-, moderate-, and low-penetrant genes alike, modifying the likelihood of cancer occurrence and its characteristics. It is very important to find the exact genetic variants causing the modifying effect at each locus and to find out what exposure is modified by this variant to be able to design appropriate preventive measures for the carriers of the genetic modifying risk factors. 

A few genes were involved at these loci. Fibroblast growth factor receptor 2 (*FGFR2*) is a mediator of signals and originates from the tissue microenvironment. It has been shown to be involved in different stages of mammary epithelial morphogenesis [24]. *FGFR2* was strongly associated with an increased risk of BC [25]. The locus on chromosome 16q12.1 had two haplotypes in this study; one was situated within the gene *TOX3* and the other one overlapped two RNA genes, one named *CASC16*. The *TOX* subfamily consists of four genes, *TOX1*, *TOX2*, *TOX3*, and *TOX4*. *TOX3* is known to be expressed in the brain. However, it was also found to be expressed at a higher level in BC than in normal tissue [26]. *CASC16* has been shown to be involved in the development and invasion of BC [27].

New disease-associated susceptibility loci have been found due to the multiallelic haplotype-based analysis approach. Haplotypes are defined as SNPs or other genetic markers that are near each other on the same chromosome and inherited together [18]. A haplotype analysis is more likely to identify founder risk alleles compared to SNP analysis. Generally, a higher OR is observed for haplotypes than for SNPs at a certain locus [10,11,12]. For identifying novel loci or variants in a homogenous population, such as the Swedish population, haplotype analysis could therefore be preferred. It can be discussed what exact *p*-value should be used for statistical significance when millions of estimated haplotypes over the genome are evaluated repeatedly as in sliding-window analysis. The *p*-value is dependent on the difference in numbers of cases and controls, which in turn is dependent on the risk at each locus. At the same time, each SNP is included in the analysis of all windows 24 bases up- and downstream, which theoretically could result in several millions of haplotypes. In practice, the number of haplotypes tested in our population was less than ten million. On the other hand, examining haplotypes over large regions also means that the same putative locus is tested numerous times and results in many haplotypes of different lengths for each locus. Many tests are performed, which could need to be adjusted for. However, numerous variations of haplotypes for the same locus occur, why they can also be considered as replications and as such would serve to adjust the *p*-value in the opposite direction. The results for chromosome 10 can serve as a good example of this. All the statistically significant haplotypes (869) represent the same locus on 10q26.13 (BP1:123338552-BP2:123338654, GRCh37) (Supplemental Table S1). In addition, the same locus came up thousands of times without statistically significant *p*-values. Thus, there is no other locus of interest on this chromosome. The second most significant locus on chromosome 10 is at BP1:34511990-BP2:34629851 (GRCh37) (OR = 1.96, *p* = 8 × 10^−7^) (Supplemental Table S1). For this study, *p* = 5 × 10^−8^ was employed for statistical significance since this is generally accepted for GWASs.

A strength of this study is the relatively large and homogenous cohort of BC patients and healthy controls from Sweden. Haplotype analysis could find both common and rare loci; however, the result is always highly dependent on what SNPs are used in the study. The Oncochip was generated with a genome-wide backbone of about 300,000 common SNPs, plus extra SNPs chosen for fine-mapping of various known risk loci from previous GWASs. This means that the chip was not designed to find all or even most of the risk loci. Still, the results were clear enough to draw some important conclusions and suggestions for future studies, in a larger Swedish population and other populations.

The risk of BC can be high in those with inherited high-risk genes, moderate in carriers of more moderate-risk genes, and modest in those with risk variants in loci suggested in analysis of sporadic, familial, and all BC cases. The ORs in all three analyses varied, and the ORs obtained in the analysis of all cases are expected to best predict the increased risk for the general population. All ORs were less than two for the five statistically significant loci presented in all BC types in all three studies. However, they were still higher than most ORs from published GWASs in BC [7,28,29,30,31,32,33,34,35,36]. Although ORs in the Swedish studies were inflated by the fact that controls were selected to have no family history for BC, they still suggest that haplotype GWASs could find other risk loci than single-variant GWAS. Moreover, the results suggest that it would be important to always study not only all cases but also sporadic and familial cases separately. A limitation of this study is that it could not be replicated since no data from other European populations were available for study.

## 5. Conclusions

A haplotype GWAS in sporadic, familial, and all Swedish BC cases supported the well-known risk loci on chromosomes 10q26.13, 11q13.3, and 16q12.1 and found one novel locus on chromosome 12p11.21 to act as a genetic modifier of BC risk. The current study suggested that haplotype GWAS analysis of sporadic, familial, and all BC cases found risk loci with ORs below two. The ORs for loci found in analysis of sporadic and all BC cases were similar, and those were therefore suggested to act as modifiers. It is important to find such risk factors to be able to design appropriate preventive measures. Haplotype analysis can find other loci, most often associated with higher risks than single-variant analysis. Further studies are needed to find out how to best implement the findings in clinical practice.

## Figures and Tables

**Table 1:** Statistically significant loci from haplotype GWAS in sporadic breast cancer.

Locus	BP1	BP2	Haplotype	F	OR	*p*	Genes
10q26.13	121579038	121579140	GG	0.4	1	1.96 × 10^−15^	*FGFR2*
11q13.3	69502113	69513996	GGAAAAGGGA	0.1	1	2.30 × 10^−9^	RNA
12p11.21	31804562	31877649	GAGGGGGGAAGCG	0.1	1	4.55 × 10^−8^	RNA
16q12.1	52552656	52563932	GGG	0.3	1	1.32 × 10^−14^	*CASC16*
16q12.1	52526301	52528899	AG	0.3	1	2.96 × 10^−14^	*TOX3*

Table showing the haplotype loci with *p*-values *p* < 5 × 10^−8^; BP1 and BP2, first and last base pairs (GRCh38) in the haplotype; F, frequency; “Haplotype” shows the genotype value for each SNP in the haplotype.

**Table 2:** Comparison of haplotypes in sporadic BC with the same haplotypes in familial and all BC.

Locus	GWAS Sporadic (n = 2550)		GWAS Familial (n = 650)		GWAS All (n = 3200)	
	OR	*p*-Value	OR	*p*-Value	OR	*p*-Value
10q26.13	1.33	1.96 × 10^−15^	1.53 **	1.70 × 10^−12^	1.36 *	1.00 × 10^−20^
11q13.3	1.39	2.30 × 10^−9^	1.66 **	2.58 × 10^−9^	1.44 *	6.37 × 10^−13^
12p11.21	1.42	4.55 × 10^−8^	1.08	0.524	1.35	7.92 × 10^−7^
16q12.1	1.34	1.32 × 10^−14^	1.45 **	4.39 × 10^−9^	1.37 *	2.38 × 10^−18^
16q12.1	1.34	2.96 × 10^−14^	1.49 **	3.97E × 10^−10^	1.37 *	1.71 × 10^−18^

This table describes the ORs and *p*-values for the statistically significant haplotypes together with the results for the same haplotypes in the analyses of familial and all samples. Loci presented in [10] * and [11] **.

## Data Availability

Access to the data is controlled. However, Swedish laws and regulations prohibit the release of individual and personally identifying data. Therefore, the whole data cannot be made publicly available. The data that support the findings of this study are available from the corresponding authors upon reasonable request.

## Supplementary Materials

The following supporting information can be downloaded at: https://zenodo.org/records/14507892 (accessed on 12 December 2024), Tables S1–S23: Results from haplotype GWAS in sporadic breast cancer for chr 1 to 23.

## Abbreviations

BC, breast cancer; GWAS, genome-wide association study; SNP, single-nucleotide polymorphism; BCAC, Breast Cancer Association Consortium; CRC, colorectal cancer; QC, quality control; MDS, multidimensional scaling; MAF, minor allele frequency; BP1/-2, base pair 1/-2; RNA, ribonucleic acid.
